# AglB, catalyzing the oligosaccharyl transferase step of the archaeal *N*-glycosylation process, is essential in the thermoacidophilic crenarchaeon *Sulfolobus acidocaldarius*

**DOI:** 10.1002/mbo3.185

**Published:** 2014-06-10

**Authors:** Benjamin H Meyer, Sonja-Verena Albers

**Affiliations:** 1 Molecular Biology of Archaea, Max-Planck Institute for terrestrial MicrobiologyKarl-von-Frisch-Strasse 10, 35043, Marburg

**Keywords:** AglB, Archaea, Crenarchaeota, *N*-glycosylation, *Sulfolobus*

## Abstract

*Sulfolobus acidocaldarius*, a thermo-acidophilic crenarchaeon which grows optimally at 76°C and pH 3, exhibits an astonishing high number of *N*-glycans linked to the surface (*S*-) layer proteins. The *S*-layer proteins as well as other surface-exposed proteins are modified via *N*-glycosylation, in which the oligosaccharyl transferase AglB catalyzes the final step of the transfer of the glycan tree to the nascent protein. In this study, we demonstrated that AglB is essential for the viability of *S. acidocaldarius*. Different deletion approaches, that is, markerless in-frame deletion as well as a marker insertion were unsuccessful to create an *aglB* deletion mutant. Only the integration of a second *aglB* gene copy allowed the successful deletion of the original *aglB*.

## Introduction

The asparagine (*N*)-linked protein glycosylation is one of the most predominant co- and posttranslational protein modifications, which is found in all three domains of life (Larkin and Imperiali 2011). In Eukarya *N*-glycosylation is an essential process, which is evolutionary highly conserved from yeast to human (Lehle et al. 2006). It is estimated that more than half of all eukaryotic proteins are glycoproteins (Apweiler et al. 1999; Zielinska et al. 2010). The biological functions of protein glycosylation span a broad spectrum, for example, as it is influencing protein folding and stability, intra and extracellular recognition, or enzyme activity, which can be crucial for the development and survival of an organism (Varki 1993; Helenius and Aebi 2004; Caramelo and Parodi 2007).

The key enzyme of the *N*-glycosylation process is the oligosaccharyl transferase (OTase). The OTase is catalyzing the last step of this process by transferring the fully assembled *N*-glycan en bloc from a lipid pyrophosphate donor onto a selected asparagine within a specific *N*-glycosylation recognition site Asn-x-Thr/Ser of a nascent protein, where x can be any residue except proline (Gavel and Vonheijne 1990).

In eukaryotes, this step is catalyzed in the endoplasmic reticulum (ER) lumen by the multimeric OTase complex. The OTase complex from *Saccharomyces cerevisiae* is composed of 8 nonidentical membrane subunits: Wbp1, Swp1, Stt3, Ost1, Ost2, Ost3 or Ost6, Ost4, and Ost5 (Kelleher and Gilmore 2006). The Stt3p subunit contains the catalytic site, however, Stt3p alone is not sufficient for the OTase processs (Yan and Lennarz 2002; Nilsson et al. 2003; Karamyshev et al. 2005). The detailed function of the other subunits of the OTase complex is not fully understood, but they are thought to regulate and influence the modification of the *N*-glycosylation sites (Lennarz 2007). For Ost3p and Ost6p, which are homologous, interchangeable subunits, it has been shown that the presence of either one of the interchangeable subunits affects glycosylation occupancy of a subset of *N*-glycosylation sites in yeast, and they specify the interaction with different translocation complexes (Schwarz et al. 2005; Schulz and Aebi 2009; Schulz et al. 2009). In contrast to the huge eukaryal heteromeric OTase complex, only the ortholog of Stt3p is needed for the OTase reaction in lower Eukarya, for example, *Leishmania major*, or in the prokaryotic system (Glover et al. 2005a; Igura et al. 2008; Hese et al. 2009).

The bacterial *N*-glycosylation process is not common, and orthologs of OTase key enzymes have been found only in a few species of delta- and epsilonproteobacteria (Nothaft and Szymanski 2010, 2013). So far *Campylobacter jejuni* is the only bacterium for which the bacterial *N*-glycosylation pathway has been studied in detail (Szymanski and Wren 2005), besides studies of *N*-glycosylation pathway in *Helicobacter pullorum* (Jervis et al. 2010) and an atypical protein *N*-glycosylation process in *Haemophilus influenza,* lacking an OTase (Gross et al. 2008).

The bacterial OTase PglB is a membrane protein, composed of an *N*-terminal segment with 9–13 predicted transmembrane domains (TD) and a soluble C-terminal periplasmic domain which comprises a highly conserved site (WWDYG) important for the activity. This topology is similar to the experimentally defined topology of the eukaryal Stt3p (Kim et al. 2005). However, only the single PglB enzyme is necessary to fulfill the *N*-glycan transfer, in contrast to the eukaryal multimeric OTase complex (Glover et al. 2005b). The periplasmic domain possesses a mixture of *α*/*β* folds, which has been observed in the crystal structures of the soluble domain of *C. jejuni* PglB (Maita et al. 2010). However, the isolated periplasmic domain of PglB was insufficient to catalyze the OTase reaction on its own, implying that the transmembrane segments are needed for the catalytic action. Indeed the isolation and the crystal structure of the full OTase from *Campylobacter lari* together with its bound acceptor peptide, revealed new insights into the molecular basis of the *N*-linked glycosylation mechanism (Lizak et al. 2011).

**Figure 1:**
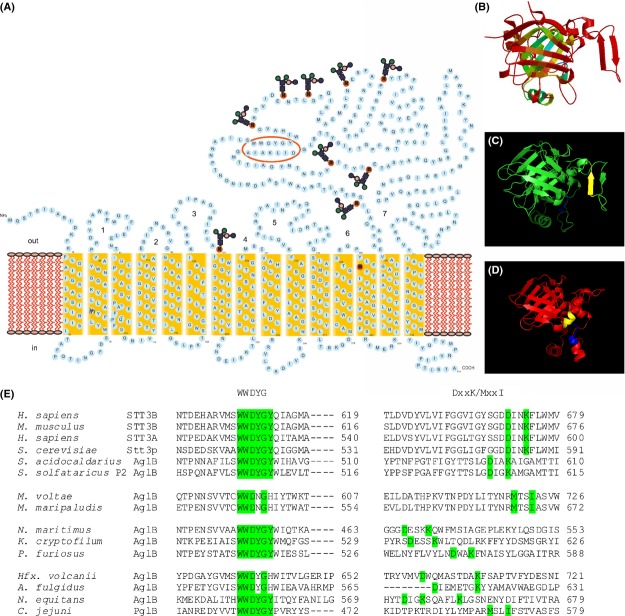
Topology model of AglB from *Sulfolobus acidocaldarius*. (A) The model is based on the TMHMM prediction server 2.0 (http://www.cbs.dtu.dk/services/TMHMM/). Transmembrane segments are indicated in yellow boxes. Asparagine residues within a predicted glycosylation site are indicated by red circle, the *N*-glycan attachment sites in *S. acidocaldarius* are not confirmed. External loops are shown in bold numbers. The conserved WWDYG and DxxK motif within the external loops 7 are circled in red. (B–D) Structural alignment of full AglB from *S. acidocaldarius* with the crystal structure of the soluble domain of AglB from *Pyrococcus furiosus* (pdb: 2zagD). (B) Alignment was performed by the SWISS-MODEL program (http://swissmodel.expasy.org/); Modeled are the residues 473–749 of the template (pdb: 2zagD)(Igura et al. 2007). Estimated per-residues inaccuracy is visualized using a color gradient from blue (more reliable regions) to red (potentially regions), indicating the low sequence identity of 17.67%. (C) Modeled structure of AglB form *S. acidocaldarius* compared with (D) the crystal structure of *P. furiosus* (AA 473–749); the conserved WWDYG and DxxK motifs are shown in blue and yellow, respectively. (E) Alignment of the WWDYG and DxxK/MxxI motifs and their flanking regions of selected OTase from the three domains of life. The amino acid sequences of the STT3, PglB, and AglB proteins were retrieved from the InterPro database, and aligned with the ClustalW program. In 2014, over 1434 sequences are grouped to the family IPR003674, including 844 Stt3, 225 PglB, and 358 AglB; in 2012, 827 sequences were grouped to this family; (530 Stt3, 96 PglB, and 201 AglB). Representative sequences from eukaryal Stt3 proteins and the bacterial PglB from *Campylobacter jejuni* were selected. The AglB sequences from the crenarchaeota *S. acidocaldarius* DSM639 and *S. solfataricus* P2; the euryarchaeota *Methanococcus voltae* strain A3, *M. maripaludis* strain A2 and *P. furiosus* DSM 3638 *Hfx. volcanii* DS2*, Archaeoglobus fulgidus* DSM4304; from the nanoarchaeota *N. equitans* strain Kin4-M; from the korachaeota K. *cryptofilum* strain OPF8; and from the thaumarchaeota *Nitrosopumilus maritimus* SCM1 were selected for the alignment. Highlighted amino acids belong to the WWDYG or DxxK/MxxI motif.

In contrast to Bacteria, the archaeal OTase AglB is found in almost all sequenced Archaea (Magidovich and Eichler 2009; Maita et al. 2010; Kaminski et al. 2013), which underlines the broad distribution of the *N*-glycosylation process and the importance of this protein modification within the Archaea. Although AglB proteins exhibit a low overall sequence homology to the Stt3 orthologs, all AglBs possess the highly conserved WWDYG motif (Magidovich and Eichler 2009; Maita et al. 2010) (Fig.1). Like the bacterial PglB and in contrast to the eukaryal Stt3p, AglB is the only enzyme needed for the OTase reaction (Igura et al. 2008). The crystal structure of the C-terminal soluble domain of AglB from *Pyrococcus furiosus* was the first structure of an OTase (Igura et al. 2007). However, like the soluble part of the bacterial PglB, the soluble part of AglB was also impaired in its function. Analyses of the crystal structure and the alignment of AglB were used to identify a second conserved motif, the DxxK, where x can be any residue (Maita et al. 2010). The crystal structure revealed that this motif lies in structural proximity to the WWDYG motif and is thought to interact with the WWDYG and coordinate the target peptide (Igura et al. 2008; Lizak et al. 2011). The importance of this motif was shown by in vivo mutational studies of the Asp and Lys residue, which showed that this motif was catalytically important in yeast and in *L. major* (Igura et al. 2008; Hese et al. 2009). The bacterial PglB is missing this DxxK motif; however, a different MxxI motif acts as the counterpart of the DxxK sequence (Maita et al. 2010; Matsumoto et al. 2012).

So far it has been demonstrated in three archaeal species that *N*-glycosylation is not essential for cell viability as AglB was successfully deleted in *Haloferax volcanii, Methanococcus maripaludis,* and *Methanococcus voltae* (Chaban et al. 2006; Abu-Qarn et al. 2007; Vandyke et al. 2009). Although the deletion of *aglB* resulted in nonmotile cells, AglB was not essential for cell growth in these euryarchaea. In this study, we have tested whether *aglB* can be deleted from the genome of the crenarchaeaon *S. acidocaldarius* and demonstrated that it is essential for viability in this organism.

## Materials and Methods

### Strains and growth conditions

The strain *Sulfolobus acidocaldarius* MW001 (Δ*pyrE*) (Wagner et al. 2012), *S. solfataricus* P2 (Zillig et al. 1980) and *S. islandicus* E233S1 (Δ*pyrEF*, Δ*lac*S) (Deng et al. 2009) and the derived mutant strains (Table1) were grown in Brock medium at 75°C, pH 3 adjusted using sulfuric acid. The medium was supplemented with 0.1% w/v NZ-amine and 0.1% w/v dextrin as carbon and energy source (Brock et al. 1972). First and second selection gelrite (0.6%) plates were supplemented with the same nutrients (as shown above), with the addition of 10 mmol/L MgCl_2_ and 3 mmol/L CaCl_2_. In addition, 10 *μ*g mL^−1^ uracil and 100 *μ*g mL^−1^ 5-fluoroorotic acid (5-FOA) were added for second selection plates. For the growth of the uracil auxotrophic mutants, 10 *μ*g mL^−1^ uracil was added to the medium. Cell growth was monitored by measuring the optical density at 600 nm.

**Table 1:** Strains and plasmids used in this study.

	Genotype	Reference
Strains		
*Sulfolobus acidocaldarius*		
MW001	*S. acidocaldarius* DSM 639, Δ*pyr*E	Wagner et al. (2012)
MW098	MW001 with *saci1162*::*aglB*	*This study*
MW099	MW001 with *ΔaglB, saci1162*::*aglB*	*This study*
*S. islandicus*		
E233S1	*S. islandicus* Rey15A, Δ*pyrEF*, Δ*lacS*	Deng et al. (2009)
Rey15A	*S. islandicus* Wild type	Contursi et al. (2006)
*S. solfataricus*		
P2	*S. solfataricus* Wild type	Zillig et al. (1980)
Plasmids		
pSAV407	Gene targeting plasmid, pGEM-T Easy backbone, *pyrEF* cassette of *S. solfataricus*	Wagner et al. (2012)
pSVA1203	In-frame deletion of *agl*B (*saci1274*) cloned into pSVA407 with *Not*I, *Bam*HI	*This study*
pSVA1204	In-frame deletion of *agl*B (*siRE1141*) cloned into pSVA407 with *Pst*I, *Bam*HI	*This study*
pSVA1241	Integration plasmid, *saci1162*::*aglB* cloned into pSVA407 with *Apa*I*, Bam*HI	*This study*
pSVA1244	*aglB*_up_-*pyrEF*-*aglB*-_down_ cloned into pUC19 with *Eco*RI, *Kpn*I	*This study*
pSVA1266	Expression plasmid of *agl3* cloned into pSVA1450 with *Nco*I, *Eag*I	Meyer et al. (2011)
pSVA1274	Expression plasmid of agl16 cloned into pSVA1450 with *Nco*I, *Eag*I	Meyer et al. (2013)

### Construction of deletion plasmids

To verify the predicted function of *aglB* participation in the last step of the *N*-glycosylation pathway, attempts to isolate a markerless deletion mutant of *aglB* in *S. acidocaldarius* MW001 and *S. islandicus* E233S1 were made using the methods previously described (Deng et al. 2009; Wagner et al. 2009, 2012). Briefly the strains MW001 and E233S1, in which the genes for the uracil biosynthesis were disrupted, were transformed with the plasmid pSVA1203 or pSVA1204, respectively (Table1). For constructing the plasmid pSVA1203, 800–1000 bp of the up- and downstream regions of *S. acidocaldarius aglB* (*saci1274*) were PCR amplified. At the 5′ end of the upstream forward primer (1725) and of the downstream reverse primer (1726) the restriction site *Apa*I and *Bam*HI were introduced, respectively (Table2). The upstream reverse primer (1712) and the downstream forward primer (1713) were designed to incorporate each 15 bp of the reverse complement strand of the other primer, resulting in a 30 bp overlapping stretch. For constructing the plasmid pSVA1204, 800–1000 bp of the up- and downstream fragments of *S. islandicus aglB* were PCR amplified. The upstream forward primer (1727) included the restriction site for *Pst*I and the reverse primer (1718) contained at its 5′end 15 bp of the reverse complement strand of the downstream forward primer. The downstream forward primer (1719) incorporated as well 15 bp of the reverse complement strand of the upstream reverse primer at its 5′end, leading to a 30 bp overlapping stretch of both internal primers. The upstream reverse primer (1728) was designed to incorporate a *BamH*I restriction site at is 5′end. All up- and downstream fragments were fused by an overlapping PCR, using the 3′ ends of the up- and downstream fragments as reverse primers.

**Table 2:** Primers used in this study.

Primer	Sequence (5′–3′)	Restriction site
*saci1162*::*aglB*		
1891	CTCACTGGGCCCGACCAAGAGCAGACAAAGAG	*Apa*I
1892	ATT TCAACAGCTTAAGGTCTAACCGTAAGGAGGTTCCTG	
1893	CCTTACGGTTAGACCTTAAGCTGTTGAAATTGTAGGTGGAATTATAAC	
1894	CTTGAATATCTATTTTTATTTATGCAAAGTACTAGTATACTGGCTAGAATTGATAAG	
1895	TATACTAGTACTTTGCATAAATAAAAATAGATATTCAAGTTTATAAATTTTTTGGTG	
1896	CGCCGAGGATCCGTCACTGAACAATATCCCCTC	*BamH*I
1713	ATGCAAAGTACTTAAGTAACTAACGCATTAGGAGGAG	
991	TTGCAGACAAGGGTATATCC	
1896	CGCCGAGGATCCGTCACTGAACAATATCCCCTC	
1706	CCCGTCGACTACGGAAATGCCTTTGACAG	
In-frame deletion of *aglB* (*saci1274*)		
1725	ATAAGAATGCGGCCGCTATTAGAATAGTAGTCACCACTAATAATC	*NotI*
1712	CTAATGCGTTAGTTACTTAAGTACTTTGCATCAGATAAGTAG	
1713	ATGCAAAGTACTTAAGTAACTAACGCATTAGGAGGAG	
1726	CCCCGCGGATCCTACGGAAATGCCTTTGACAG	*BamH*I
In-frame deletion of *aglB* (*siRE1141*)		
1727	CCCACTCTGCAGTCACTTGGAGGGTAAGAG	*Pst*I
1718	AACACCGGGCTGTGTTTACTTAACTGACTGCATGGCTGTAG	
1719	ATGCAGTCAGTTAAGTAAACACAGCCCGGTGTTATAC	
1728	CCCCGCGGATCCAAAGATGCGTGGGAGCAAG	*BamH*I
*aglB*::*pyrEF*		
1802	CCCACTGAATTCAACACCCGAGGTAAGAGATACAC	*EcoR*I
1803	CTCAAACCTTTAAGGGAACGCAGTAATGCTAATG	
1804	TTCCCT TAAAGGTTTGAGCAGTTCTAGTACTTG	
1783	GTTAGTTACGGAGGGATCCGACCGGCTATTTTTTCAC	
1784	CGGTCGGATCCCTCCGTAACTAACGCATTAGGAGGAG	
1785	CCCACTGGTACCAAGTTCCCATCAGACGGAGAAG	*Kpn*I

For constructing the plasmid pSVA1241, used for the deletion of *saci1162,* encoding an *α* amylase, by the integration of *aglB* (saci1274)*,* the upstream region of *saci1162*, the full-length *aglB,* and downstream region of *saci1162* were amplified with the following primer 1891 + 1892, 1893 + 1894, and 1895 + 1896, respectively. At the 5′ end of the upstream forward primer (1891) and of the downstream reverse primer (1896), the restriction site *Apa*I and *Bam*HI were introduced, respectively. The upstream reverse primer (1892) and the *aglB* forward primer (1893) were designed to incorporate each 15 bp of the reverse complement strand of the other primer, resulting in a 30 bp overlapping stretch. The *aglB* reverse primer (1894) and the downstream forward primer (1895) were designed to incorporate each 21 bp of the reverse complement strand of the other primer, resulting in a 42 bp overlapping stretch. The upstream, *aglB*, and downstream fragments were fused by an overlapping PCR, using the 3′ ends of each fragments as primers.

The overlap PCR fragments were purified and digested with *Apa*I and *Bam*HI (Δ*saci1274 and* (*aci1162*::*aglB*), or *Pst*I and *BamH*I (Δ*sire1141*) and ligated in the predigested plasmid pSVA407, containing *pyrEF* (Wagner et al. 2012). The obtained deletion plasmids pSVA1203 (Δ*saci1274*), pSVA1204 (Δ*sire1141*), and pSVA1241 (*Saci1162*::*aglB)* were transformed into *E. coli* DH5*α* and selected on LB-plates containing 50 *μ*g mL^−1^ ampicillin. The accuracy of the plasmids was ascertained by sequencing. In order to avoid restriction in *S. acidocaldarius* and *S. islandicus* the plasmids were methylated by transformation in *E. coli* ER1821 cells containing pM.EsaBC4I (available from NEB), which expresses a methylase.

### Constructing the plasmid for the linear *aglB*_up_-*pyrEF*-*aglB*_down_ fragment integration

In order to further underline the essential property of *aglB* in *S. acidocaldarius,* a disruption of the *aglB* gene by the direct homologous integration of the *pyrEF* cassette was performed. For this approach, 1200 bp of the *aglB* upstream region, the full 1525 bp of the *pyrEF* cassette, and 1061 bp of the *aglB* downstream region were PCR amplified. For the amplification of the *aglB* upstream region the upstream forward primer (1802) incorporated a *Eco*RI restriction site, whereas the upstream reveres primer (1803) incorporated 15 bp of the reverse complementary strand of the *pyrEF* forward primer. The *pyrEF* forward primer (1804) incorporates 15 bp of the reverse complementary strand of the upstream reveres primer, leading to a 30 bp overlapping stretch. The *pyrEF* reverse primer (1783) was designed to incorporate 15 bp of the reverse complementary strand of the downstream forward primer. The *aglB* downstream forward primer (1785) incorporated 15 bp of the reverse complementary strand of the *pyrEF* reverse primer at it 5′end. The *aglB* downstream reverse primer (1785) possesses a *Kpn*I restriction site at the 5′end. The three PCR fragments were fused via an overlap PCR using the 3′ overlapping ends of each PCR fragment. The amplified 3765 bp overlap PCR fragment was further amplified using the outer primers (1802 and 1785), digested with *Eco*RI and *Kpn*I and ligated into the pUC19 vector, predigested with the same restriction enzymes. The obtained plasmid pSVA1244 (*aglB*_up_-*pyrEF*-*aglB*_down_) was transformed into *E. coli* DH5*α* and selected on LB-plates containing 50 *μ*g mL^−1^ ampicillin. The accuracy of the plasmid was verified by sequencing. Before transformation in *S. acidocaldarius* the plasmid was digested with *Kpn*I and *Eco*RI, to create the linear *aglB*_up_-*pyrEF*-*aglB*_down_ fragment.

### Transformation and selection of the deletion mutant in *S. acidocaldarius*

Generation of competent cells was preformed based on the protocol of Kurosawa and Grogan (Kurosawa and Grogan 2005). Briefly *S. acidocaldarius* strain MW001 was grown until an OD_600_ between 0.1 and 0.3 in Brock medium supplemented with 0.1% w/v NZ-amine and 0.1% dextrin. Cooled cells were harvested by centrifugation (2000*g* at 4°C for 20 min). The cell pellet was washed three times in 50 mL, 10 mL, and 1 mL of ice cold 20 mmol/L sucrose (dissolved in demineralized water) after mild centrifugation steps (2000*g* at 4°C for 20 min). The final cell pellet was resuspended in 20 mmol/L sucrose to a final OD_600_ of 10 and stored in 50 *μ*L aliquots at −80°C. 400–600 ng of methylated pSVA1203, pSVA1204, pSVA1241 plasmids or the linearized *ablB*_up_-*pyrEF*-*aglB*_down_ fragment was added to the 50 *μ*L aliquot of competent MW001 cells and incubated for 5 min on ice, before transformation in a 1 mm gap electroporation cuvette at 1250 V, 1000 Ω, 25 mF using a Biorad gene pulser II (Biorad, München, Germany). Directly after transformation 50 *μ*L of a 2x concentrated recovery solution (1% sucrose, 20 mmol/L beta-alanine, 20 mmol/L malate buffer pH 4.5, 10 mmol/L MgSO_4_) was added to the sample and incubated at 75°C for 30 min under mild shaking conditions (150 r.p.m.). Before plating, the sample was mixed with 100 *μ*L of heated 2× concentrated recovery solution and two aliquots of 100 *μ*L were spread onto two different gelrite plates containing Brock medium supplemented with 0.1% NZ-amine and 0.1% dextrin. After incubation for 5–7 days at 75°C large brownish colonies were used to inoculate 50 mL of Brock medium containing 0.1% NZ-amine and 0.1% dextrin, each culture was incubated for 3 days of 78°C. Each culture, which has been confirmed by PCR to contain the genomically integrated plasmid, was grown in Brock medium supplemented with 0.1% NZ-amine and 0.1% dextrin until an OD of 0.4. Aliquots of 40 *μ*l were spread on second selection plates, supplemented with 0.1% NZ-amine and 0.1% dextrin and 10 *μ*g mL^−1^ uracil, were incubated for 5–7 days at 78°C. Newly formed colonies were streaked out on new second selection plates to ensure that they were formed from single colonies, before each colony was screened for the genomic absence, presence or modification of the *aglB* gene by PCR.

## Results

### *S. acidocaldarius aglB* is localized next to genes encoding the translation machinery and separated from genes coding for GTases

The OTase AglB in *S. acidocaldarius*, encoded by *saci1274,* was first identified by bioinformatic methods, studying the distribution of the OTase among Archaea (Magidovich and Eichler 2009). AglB is a conserved membrane protein with 22% sequence identity to the eukaryal Stt3p from *S. cerevisiae*, 20% sequence identity to the bacterial PglB from *C. jejuni*, and 22% sequence identity to the archaeal AglB from *P. furiosus*. Apart from this low sequence homology, the protein possesses a similar topology with other identified OTases. Saci1274 possesses 15 predicted TD and a soluble C-terminal domain, which is enclosed between the TD 14 and 15. The eukaryal Stt3p contains 11 predicted TD and a soluble C-terminal domain (Kim et al. 2005), whereas the bacterial PglB possesses 13 *N*-terminal TD and C-terminal periplasmic domain (Lizak et al. 2011). Furthermore, the highly conserved WWDYG motif and the DxxK motif were found within the protein sequence of Saci1274 (Fig.1), which strengthens the proposed function of Saci1274 as an OTase (Magidovich and Eichler 2009; Kaminski et al. 2013).

**Figure 2:**
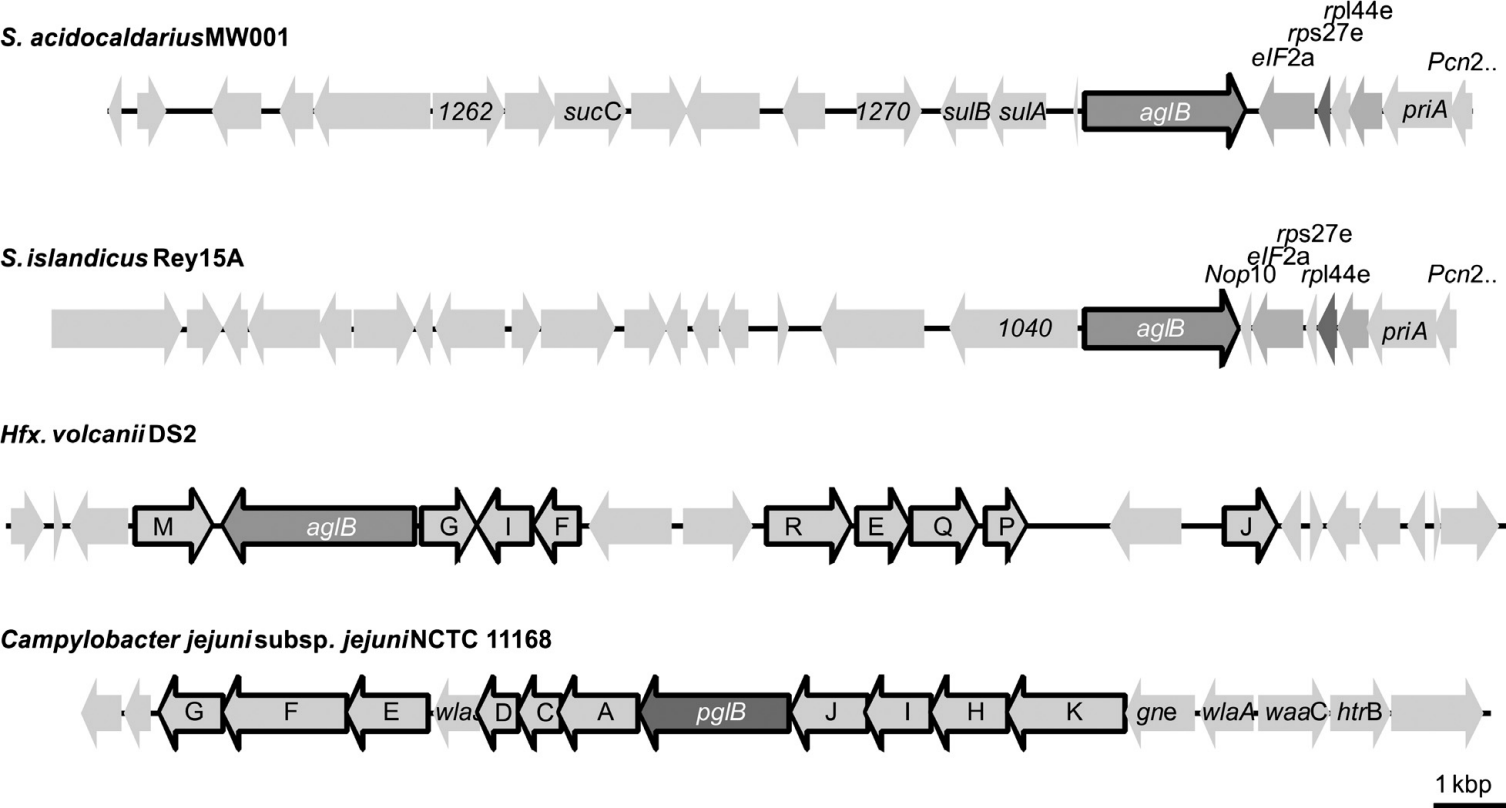
A Genetic neighborhood of the archaeal and bacterial gene coding for OTases. Physical map of the gene region of *Sulfolobus acidocaldarius* MW001, *S. islandicus* Rey15 A, *Hfx. volcanii* DS2, and *Campylobacter jejuni* in which the gene coding for oligosaccharyl transferase is located. Illustrated are the genes *saci1257* until *saci1280*, *sire_1024* till *sire_1048*, *hvol1523* till *hvol1548*, and the *Campylobacter jejuni* subsp*. jejuni* NCTC 11168 *pgl* gene cluster. Dark gray displayed genes encode the archaeal or bacterial OTase *agl*B or *pgl*B, respectively. Framed genes code for GTase or other proteins involved in the *N*-glycosylation process. Upstream of *aglB* from *S. acidocaldarius* MW001 the genes *sulA* and *sulB* encoding the sulfolobicins are found. Downstream of the *aglB* from *S. acidocaldarius* MW001 and *S. islandicus* Rey15A genes coding for the translational machinery are located.

In contrast to the gene organization of *C. jejuni* and *Hfx. volcanii*, for which the gene coding for the OTase is found directly located next to genes coding for GTases participating in the *N*-glycosylation process, no genes coding for a GTase were located directly near to *aglB* (*saci1274*) (Fig.2), as previously reported (Magidovich and Eichler 2009; Kaminski et al. 2013). Only a newly identified GTase (Saci1262) is found with distance of a few genes to *aglB* (Fig.2), which shows homologies to MurG, an *N*-acetylglucosamine transferase.

### Deletion of *aglB* by markerless in-frame deletion was unsuccessful, supporting an essential role of AglB in *S. acidocaldarius* and *S. islandicus*

**Figure 3:**
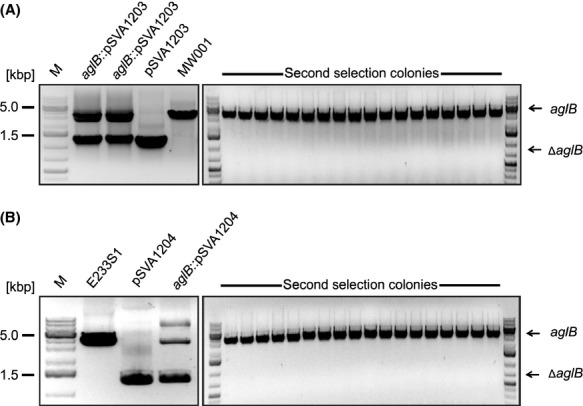
Confirmation of the integration and segregation of the *agl*B deletion plasmid pSVA1204 or pSVA1204 in *Sulfolobus acidocaldarius* MW001 and *S. islandicus* E2331*,* respectively. (A) First panel: The integration of the deletion plasmid pSVA1203 in the gene *aglB* of *S. acidocaldarius* MW001 (*aglB*::pSVA1203) was monitored by PCR using the outer primers of the upstream and downstream region of *aglB* and the genomic DNA from two-first selection colonies (*aglB*::pSVA1203). DNA from the background strain MW001 and the plasmid pSVA1203 were used as control, showing a PCR fragment corresponding to the flanking region including or excluding the *aglB* gene, respectively. Second panel: The segregation of pSVA1203 (second selection) was confirmed by PCR using the outer primers of the flanking region of *aglB* and the genomic DNA from second selection colonies. All PCR fragments gained from genomic DNA of second selection colonies correspond to the full-length *aglB* gene (3500 bp). (B) First panel: Integration of the *aglB* deletion plasmid pSVA1204 in *S. islandicus* E2331 (*aglB*::pSVA1204) was confirmed by PCR using the outside primers against the flanking regions of *agl*B and DNA isolated from one-first selection colony (*aglB*::pSVA1204). DNA isolated from the background strain E2331 or plasmid pSVA1204 were used as controls. Second panel: The segregation of the pSVA1204 (second selection) was monitored using the same flanking primer and the genomic DNA from second selection colonies. All PCR products have the calculated size of 3500 bp corresponding to the flanking regions including the full-length *aglB* gene.

Due to the low sequence similarity to the known OTase, we wanted to demonstrate the predicted function of AglB in vivo with a markerless deletion mutant of *aglB* in the genome of *S. acidocaldarius*. The *aglB* deletion mutant should be impaired in the final step of glycosylation process, resulting in the nonglycosylation of known glycoproteins, as it was shown for *Hfx. volcanii, M. voltae, and M. maripaludis* (Chaban et al. 2006; Abu-Qarn et al. 2007; Vandyke et al. 2009). The genomic integration by homologous recombination of the plasmid pSVA1203 via either the up- or downstream region of the *aglB* and the selection *pyrEF* genes was confirmed by PCR using isolated genomic DNA of strains grown on the first selection plates and the external primers of the up- and downstream region (Fig.3A). The two amplified fragments correspond to the full-length *aglB* (3500 bp) and the Δ*aglB* (1280 bp) PCR products (Fig.3A). The DNA from the background strain MW001 and the plasmid pSVA1203 were used as a PCR control, showing either the full-length *aglB* or the Δ*aglB* PCR fragment. A second recombination step, resulting in the loop out of the integrated plasmid, would produce colonies containing either the wild type genome sequence or the deleted version (Wagner et al. 2009, 2012). Screening of more than 200 second selection colonies by PCR did not reveal any Δ*aglB* strain (Fig.3A). Furthermore, the modification of the second selection condition; for example, a higher pH of 4 or a reduced incubation temperature of 60°C, did not lead to the preferred deletion strain. This result indicated that *aglB* might be essential in *S. acidocaldarius*.

To test whether we were not able to obtain any *aglB* mutant in *S. acidocaldarius* due to a problem caused by alterations in the genetic neighborhood, we wanted to create a markerless *aglB* deletion mutant in the *S. islandicus* strain E233S1 (Deng et al. 2009). *S. islandicus* shows a different *aglB* upstream gene organization, lacking the gene coding for the tRNA-Ala (Fig.2). The genomic integration of the plasmid pSVA1204, incorporating only the up- and downstream regions of *aglB* and the selection genes *pyrEF*, was confirmed by PCR (Fig.3B). The DNA from the background strain *S. islandicus* E233S1 and the plasmid pSVA1204 were used as a PCR control. A PCR performed with genomic DNA from the strain, with plasmid pSVA1204 incorporated into the genome, generated two PCR fragments corresponding to the up- and downstream region containing either the full-length *aglB* or Δ*aglB*. Screening of 100 sec selection colonies for the presence of the Δ*aglB* version, revealed only the presence of strains possessing the full-length *aglB* wild type version (Fig.3B), as it was shown for *S. acidocaldarius* (Fig.3A). This result further strengthens the idea that the oligosaccharyltransferase AglB is essential for the viability and survival of *Sulfolobus* species.

### Disruption of *aglB* by the insertion of the *pyrEF* selection marker resulted in a lethal phenotype

As the procedure of markerless in-frame deletion resulted in any *aglB* deletion mutants, we wanted to enforce the deletion of *aglB* by a single homologous recombination step disrupting the *aglB* gene by the insertion of the *pyrEF* selection cassette. Furthermore, the PCR fragment of the upstream region of *aglB* was changed leaving the first 163 bp on the *aglB* untouched, thereby avoiding the interference with the transcription start of the tRNA-Ala, found directly upstream of *aglB*. The linearized *aglB*_up_-*pyrEF*-*aglB*_down_ fragment was transformed into competent MW001 cells, cells were subsequently streaked on a first selection plate. After 9 days of incubation only tiny colonies were detected on the first selection plate. However, none of these colonies were able to grow in liquid first selection medium or on a second first selection plate. This result showed that the direct disruption of *aglB* leads to a lethal phenotype, which furthermore strengthens the idea that *aglB* is essential for the viability of *S. acidocaldarius*.

### Successful deletion of *aglB* in a *S. acidocaldarius saci1162*::*aglB* background strain

**Figure 4:**
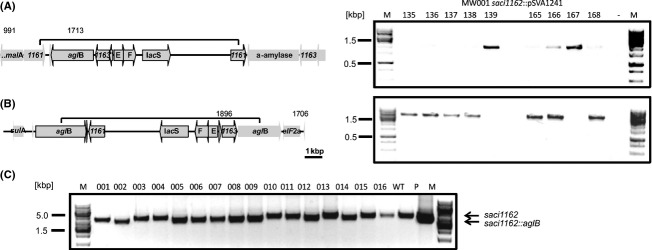
Genomic integration of a second *aglB* gene copy in the *saci1162* locus. (A) Left panel: Physical map of the homologous integration of pSVA1241 into the genome of *Sulfolobus acidocaldarius* via the *saci1162* upstream region. Integrated plasmid is indicated by the black line above the genes. Right panel: PCR using a forward primer binding upstream of the integration site and a reverse primer binding to an internal plasmid site (primer binding sites are indicated in the physical map as arrows) confirmed the integration of the plasmid via the upstream region of the colonies 139 and 167. (B) Left panel: Physical map of the integration of pSVA1241 via the *aglB* region in to the genome of *S. acidocaldarius*. Integrated plasmid is indicated by the black line above the genes. PCR, using primer binding to an internal plasmid region and to the downstream *aglB* region (primer binding sites are indicated in the physical map as arrows), confirmed the integration directly with *aglB* of the colonies 135-138 and 165, 166, and 168. (C) The segregation of the plasmid pSVA1241 was confirmed by PCR using primers binding outside of the *saci1162* flanking regions on genomic DNA derived from second selection colonies. PCR products correspond either to the wild type *saci1162* (4337 bp) or the *saci1162*::*aglB* mutants (3977 bp). Plasmid pSVA1241 (P) used for the homologous recombination and the genomic DNA of the wild type strain (WT) were used as a control.

So far every attempt to create a deletion mutant of *aglB* failed. These attempts included the markerless in-frame deletion procedure at low temperature (60°C) as well as a direct homologous recombination with *pyrEF* disrupting the *aglB* gene. All these attempts hinted at an essential role of AglB in *S. acidocaldarius*. To confirm the essential properties of AglB, a second copy of *aglB* was integrated in exchange for *saci1162*, encoding an *α*-amylase, which has been shown to be not essential in *S. acidocaldarius* (Worthington et al. 2003; Gristwood et al. 2012). The integration of the plasmid pSVA1241, used for the homologous recombination and integration of the second *aglB* gene copy, can occur in different ways (i) via the upstream *aglB* region (Fig.4A), (ii) the downstream *aglB* region or (iii) directly by the *aglB* region (Fig.4B). To confirm upstream integration of the plasmid pSVA1241, a PCR was performed using a forward primer against the upstream region of the saci1162 (Primer 991) and a reverse primer against the internal region of *aglB* (Primer 1713) (Fig.4A). Furthermore, the homologous recombination of the plasmid via the *aglB* gene was tested by a PCR using a forward primer against *pyrEF* cassette (Primer 1896) and a reverse primer against the downstream region of the original *aglB* (Primer 1706) (Fig.4B). The PCR result showed that the colonies 139 and 167 have integrated the plasmid pSVA1241 via a homologous recombination of the upstream region of the *α*-amylase (*saci1162::*pSVA1241), as it is shown in Figure4A. For other tested colonies, the PCR using a forward primer binding to the internal site of the plasmid and a reverse primer binding to the downstream region of *aglB* showed that integration of the plasmid occurred via the *aglB* region, within the colonies 135–138, 165, 166, and 168 (Fig.4B). The fact that most of the selected strains showed integration directly via the *aglB* site, is most likely due to the larger size of *agl*B compared to the *α*-amylase upstream region. However, none of the selected colonies showed an integration of the downstream region.

**Figure 5:**
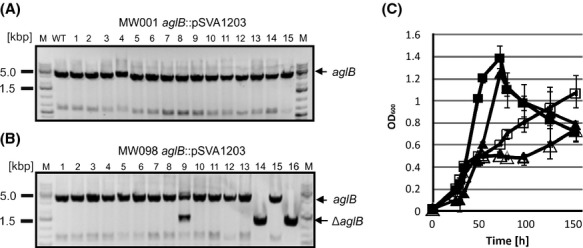
Confirmation and growth of the MW099 (Δ*aglB, saci1162*::*aglB*) strain. Colony PCR using second selection colony derived from the strains integrated the deletion plasmid pSVA1203 in the *aglB* site: MW001*aglB*::pSVA1203 strain (A) or the MW098 *aglB*::pSVA1203 strain (B) with the forward primer against the upstream region and the reverse primer against the downstream region of *agl*B revealed the presence of Δ*agl*B PCR fragments only in the colonies 14 and 16 derived from the MW098 *aglB*::pSVA1203 strain. None Δ*agl*B PCR fragment could be detected in the MW001 *aglB*::pSVA1203 background strain. (C) Growth of the MW001 background strain (rectangle) and the MW099 (Δ*aglB*, *saci1162*::*aglB*) strain (triangles) in Brock medium supplemented with 0.1% xylose (filled symbols) or 0.1% maltose (open symbols) were measured by the optical density at 600 nm.

The colony 167, in which the plasmid integrated via the *α*-amylase upstream region (*saci1162::*pSVA1241), was used for second selection. Colony PCR, using forward and reverse primer outside of the *α*-amylase (*saci1162*) region with genomic DNA from obtained second selection colonies, showed that the colonies 002, 005-009, 012, and 014 successfully integrated the second copy of *aglB* by replacing the *saci1162* gene (Fig.4C). These colonies showed the calculated PCR fragment size of 3977 bp corresponding to the *saci1162*::*aglB* mutant region, as it is shown in the plasmid control (Fig.4C). The colony 002, in which the *α*-amylase was substituted by *aglB* (*saci1162*::*aglB*), was termed strain MW098 and used as a background strain to create an *aglB* knockout of the original gene site. First selection colonies of MW001 and MW098 (s*aci1162*::*aglB*) transformed with the *aglB* deletion plasmid pSVA1203 were confirmed by PCR (data not shown), and used for second selection procedure. Selected colonies were screened for the presence of an *aglB* deletion mutant. PCR revealed two colonies (colony 14 and 16) originating from the MW098 (s*aci1162*::*aglB*) background strain which showed the calculated PCR fragment size of 1280 bp corresponding to the Δ*aglB* region (Fig.5B). The colony 16 was selected as the new strain MW099 (Δ*aglB, saci1162*::*aglB*). However, none of the 40 newly selected colonies in the MW001 background showed any fragment corresponding to the Δ*aglB* region by PCR amplification, which showed only the PCR product corresponding to the full *aglB* (3500 bp) (Fig.5A), as it was shown before (Fig.3A). The results highlight that the difficulty generating a Δ*aglB* mutant in a MW001 background does not originate from the genetic approach, in which the recombination might cause lethal polar effects. The fact that this plasmid can only be used in a *saci1162*::*aglB* background to create the Δ*aglB* mutant, clearly demonstrates that *aglB* is essential for the survival of *S. acidocaldarius*.

### Deletion of *aglB* in the *saci1162*::*aglB* background strain was not altered under noninduced conditions

Since the second copy of the *aglB* is under control of the *α*–amylase promoter, which is maltose inducible, we hypothesize that growth of MW099 (Δ*aglB saci1162*::*aglB*) is maltose dependent. For this reason the MW001 and the MW099 were grown in Brock medium supplemented with 0.1% maltose or 0.1% xylose (Fig.5C). In medium containing xylose MW099 had a longer lag phase than MW001, but reached the same final OD (Fig.5C). It seems that the basal transcription levels of the *amyA* promoter under noninduced conditions are sufficient for optimal aglB expression. In Brock medium supplemented with 0.1% maltose, the growth of the mutant as well as MW001 was reduced compared to the medium supplemented with 0.1% xylose. MW099 stopped growing at an OD_600_ of 0.6, but after a lag phase of around 50 h reached the same final OD as the MW001 background strain. We believe that the 15-fold induction of the amyA promoter under induced conditions (Wagner et al. 2014), might lead to an overexpression of AglB causing the growth defect by influencing membrane organization.

## Discussion

The OTase (Stt3p/AglB/PglB) as the key enzyme of the *N*-glycosylation process has to regulate and select glycosylation of sequences, thereby catalyzing the en bloc transfer of the lipid linked oligosaccharide onto a nascent protein. For this it has to interact with the translocon as well as with the subsequent protein folding process. To achieve this, higher Eukarya evolved a multimeric Otase complex, which is not only supporting but also essential for the OTase activity. Based on the lack of an *N*-glycosylation process in bacterial model organism *Escherichia coli* it was assumed that this co- and posttranslational modification is restricted to Eukarya. As so the discovery of a prokaryotic *N*-glycosylation process in Archaea as well as in a few bacterial species, depending on a single AglB or PglB Otase subunit homologous to the eukaryotic Stt3p, was unexpected (Szymanski et al. 1999; Wacker et al. 2002; Weerapana and Imperiali 2006; Maita et al. 2010).

In contrast to the essential properties of the eukaryal *N*-glycosylation, the prokaryotic *N*-glycosylation process can be abolished by the deletion of *aglB* or *pglB* (Wacker et al. 2002; Chaban et al. 2006; Abu-Qarn et al. 2007; Vandyke et al. 2009). In *C. jejuni,* the deletion of *pglB* did not lead to a drastic change of the phenotype, although more than 65 proteins with a multitude of cellular functions have been shown to be modified via *N*-glycosylation (Scott et al. 2011). The most important effect of the deletion of *pglB* is the nonglycosylation of the type 4 secretion system (T4SS), which effected animal colonization and invasion (Szymanski and Wren 2005). In the archaeal species, *Hfx. volcanii*, *M. voltae, and M. maripaludis*, for which the *N*-glycosylation process has been studied in detail, the deletion of *aglB* does not influence growth under standard growth conditions (Chaban et al. 2006; Abu-Qarn et al. 2007; Vandyke et al. 2009). However, the deletion of *aglB* in each of these archaeal species results in an non glycosylated archaellum, as the cells appear nonarchaellated and are impaired in their motility (Chaban et al. 2006; Abu-Qarn et al. 2007; Vandyke et al. 2009; Tripepi et al. 2012). Furthermore, the depletion of the *N*-glycosylation process in *Hfx. volcanii* has an effect on the growth under elevated salt concentration as well as an enhanced release of the *S*-layer glycoproteins into the medium (Abu-Qarn et al. 2007). However, the nonessential property of *aglB* in the so far studied archaeal species is in contrast to our findings, in which we demonstrated that *aglB* is essential for the viability of *S. acidocaldarius*. The essentiality of *aglB* from *S. acidocaldarius* might reflect a stronger need for the thermostabilization of proteins by the *N*-glycosylation process. Indeed the number of glycosylation sites found in mesophilic and thermophilic archaeal *S*-layer proteins underlines this idea (Meyer and Albers 2013). In the mesophilic and halophilic archaeon *Hfx. volcanii,* seven glycosylation sites are predicted within the *S*-layer amino acid sequence (Jarrell et al. 2010), of these six sites have been experimentally shown to be modified *N*-glycans (Sumper et al. 1990; Mengele and Sumper 1992; Abu-Qarn et al. 2007; Magidovich et al. 2010; Parente et al. 2014). In contrast, more than 30 glycosylation sites could be detected within the amino acid sequence of the *S*-layer glycoprotein from the thermophilic *S. acidocaldarius* and other species from the *Sulfolobales*. Of the eleven *N*-glycosylation sites analyzed in the C-terminal part of the *S*-layer protein, nine were experimentally confirmed to be modified, whereas the other two sites are likely to be glycosylated (Peyfoon et al. 2010). This high amount of *N*-glycosylated residues, where one modification is found after an average stretch of 30–40 residues, has not been reported for any other Archaea, however, it seems that thermo(acido)philic Archaea tend to possess *S*-layer proteins with a remarkably high number of predicted *N*-glycosylation sites compared to mesophilic Archaea.

The discrepancy between the *N*-glycosylation frequencies in different Archaea might explain why the *N-*glycosylation process can be abolished in *Hfx. volcanii, M. maripaludis* or *M. voltae,* as these organisms show only a minor amount of glycosylation modification of the *S*-layer protein (Meyer and Albers 2013). Indeed possessing fully stable *S*-layer, as the sole cell envelope of *S. acidocaldarius*, is of great importance for the cell integrity and viability of this organism. Defects in the biosynthesis of the full-length *N*-glycan in the MW039 (Δ*agl3*) and MW043 (Δ*agl16*) deletion strains of *S. acidocaldarius* show that the growth (under high salinity) as well as the motility is strongly dependent on the *N*-glycan size (Meyer et al. 2011, 2013). These results demonstrated that even one missing hexose of the *N*-glycan has an effect on the growth and motility. These effects were increased with the reduction in the amount of the *N*-glycan sugars (Meyer et al. 2011, 2013).

The essentiality of the *N*-glycosylation process in *S. acidocaldarius* was furthermore supported by the use of the antibiotics bacitracin (Meyer and Schafer 1992) and tunicamycin (Hjort and Bernander 1999). The treatment with these antibiotics led to cell growth arrest and later cell death of *S. acidocaldarius*, as these antibiotics interfere with the initial steps of the *N*-glycosylation. Bacitracin blocks the release of one phosphate from dolichol pyrophosphate, thereby blocking the regeneration of dolichol phosphate, used as the *N*-glycan lipid carrier. Tunicamycin inhibits the initiation step of the *N*-glycosylation by blocking the active site of UDP-*N*-acetylglucosamine-1-phosphate:dolichyl-phosphate GlcNAc-1-phosphotransferase (Alg7 in Eukaria, AglH in Archaea). However, it should be mentioned that in contrast to the deletion of *aglB* the deletion of *aglH* homologs in *M. voltae* (Chaban et al. 2006) and *M. maripaludis* (D. VanDyke and K. F. Jarrell, unpublished data) were also unsuccessful, implying the essentiality the lipid modification with glycans, beyond the *N*-glycosylation.

The advantage of having now an expression system in Sulfolobus (Wagner et al. 2012) will lead to future experiments elucidating the catalytic mechanism of AglB, and will provide us with new insights about AglB, that is, glycosylation state, interaction partners, selectivity of substrate, and temperature activity.
